# Combined high-resolution genotyping and geospatial analysis reveals modes of endemic urban typhoid fever transmission

**DOI:** 10.1098/rsob.110008

**Published:** 2011-10

**Authors:** Stephen Baker, Kathryn E. Holt, Archie C. A. Clements, Abhilasha Karkey, Amit Arjyal, Maciej F. Boni, Sabina Dongol, Naomi Hammond, Samir Koirala, Pham Thanh Duy, Tran Vu Thieu Nga, James I. Campbell, Christiane Dolecek, Buddha Basnyat, Gordon Dougan, Jeremy J. Farrar

**Affiliations:** 1 The Hospital for Tropical Diseases, Wellcome Trust Major Overseas Programme, Oxford University Clinical Research Unit, 190 Ben Ham Tu, Quan 5, Ho Chi Minh City, Vietnam; 2 Oxford University Clinical Research Unit, Patan Academy of Health Sciences, Kathmandu, Nepal; 3 Department of Microbiology and Immunology, The University of Melbourne, Melbourne, Australia; 4 The Wellcome Trust Sanger Institute, Hinxton, Cambridge, UK; 5 University of Queensland, School of Population Health, Brisbane, Australia; 6 The MRC Centre for Genomics and Global Health, Oxford, UK

**Keywords:** Salmonella, Typhoid, Paratyphoid, genotyping, transmission, geospatial

## Abstract

Typhoid is a systemic infection caused by *Salmonella* Typhi and *Salmonella* Paratyphi A, human-restricted bacteria that are transmitted faeco-orally. *Salmonella* Typhi and *S*. Paratyphi A are clonal, and their limited genetic diversity has precluded the identification of long-term transmission networks in areas with a high disease burden. To improve our understanding of typhoid transmission we have taken a novel approach, performing a longitudinal spatial case–control study for typhoid in Nepal, combining single-nucleotide polymorphism genotyping and case localization via global positioning. We show extensive clustering of typhoid occurring independent of population size and density. For the first time, we demonstrate an extensive range of genotypes existing within typhoid clusters, and even within individual households, including some resulting from clonal expansion. Furthermore, although the data provide evidence for direct human-to-human transmission, we demonstrate an overwhelming contribution of indirect transmission, potentially via contaminated water. Consistent with this, we detected *S*. Typhi and *S*. Paratyphi A in water supplies and found that typhoid was spatially associated with public water sources and low elevation. These findings have implications for typhoid-control strategies, and our innovative approach may be applied to other diseases caused by other monophyletic or emerging pathogens.

## 2 Introduction

The bacterial pathogens *Salmonella* Typhi (*S*. Typhi) and *Salmonella* Paratyphi A (*S*. Paratyphi A) are the causative agents of the classical human systemic infection known as typhoid fever [1,2]. Globally, there are an estimated 27 million cases of typhoid annually [3]. While sporadic cases involving travellers occasionally occur in developed countries [4], the vast majority of disease occurs in areas with poor sanitation in developing countries [3]. The agents of typhoid are transmitted faeco-orally and shed acutely during an infection. Additionally, some individuals, estimated to be around 5 per cent of cases, may progress to become long-term asymptomatic carriers, as both *S*. Typhi and *S*. Paratyphi A can colonize and survive for prolonged periods within the gall bladder [5,6]. A classic example of a typhoid carrier is of a cook in New York, who in the early part of the twentieth century became infamously known as Typhoid Mary [7]. Individuals like Typhoid Mary can unknowingly excrete the causative bacteria into the local environment indefinitely, representing individual reservoirs of infection that may, potentially, maintain the local pathogen population.

Typhoid is transmitted through direct contact with individuals shedding the bacteria, either during acute infection or asymptomatic carriage. Additionally, as typhoid is associated with areas of poor sanitation, indirect transmission via the consumption of contaminated food or water evidently plays an important role [8]. However, our general understanding of typhoid-transmission patterns is poor, founded mainly on the observation of declining incidence following improvements in sanitation [9,10], or through risk factors identified by classical epidemiological studies [11–13]. Yet previous epidemiological studies concerning endemic typhoid have not included a thorough molecular examination of the pathogens or a spatio-temporal investigation of infection sites in a single setting. Therefore, potential transmission events contributing to endemic disease have never been elucidated at macro- or micro-scale, and the relative influences of indirect and direct transmission and the relationship between *S*. Typhi and *S*. Paratyphi A in endemic urban areas are not understood.

A general lack of comprehensive pathogen characterization in typhoid studies is related to the fact that both *S*. Typhi and *S*. Paratyphi A are monophyletic clades within the bacterial species *Salmonella enterica*. These two genetically distinct organisms entered the human population relatively recently and have undergone evolutionary convergence to cause indistinguishable diseases [14–17], frequently occurring together in the same locations [18]. Both *S*. Typhi and *S*. Paratyphi A exhibit exceptionally low levels of genetic variation, and the current gold standard for bacterial genotyping, multi-locus sequence typing (MLST), has insufficient resolution for distinguishing within populations of these pathogens [16]. Alternative techniques such as pulsed field gel electrophoresis (PFGE) may be used to discriminate between some isolates, but do not generate robust phylogenetic information that can be applied to study global evolution or local transmission patterns [19]. However, we have recently developed single-nucleotide polymorphism (SNP) genotyping to unequivocally resolve the global *S*. Typhi population in the form of a highly parsimonious phylogenetic tree [17,20]. Using such high-resolution genotyping enables us to accurately and consistently distinguish among *S*. Typhi circulating within localized human populations, allowing the diversity of the organism to be precisely defined within any given temporal or geographical boundary [21–24].

Kathmandu, the capital city of Nepal, is a typical example of a densely populated South Asian urban setting where typhoid, caused by both *S*. Typhi and *S*. Paratyphi A, is endemic [18,25]. In an attempt to improve our understanding of the dynamics of transmission of typhoid, we have taken a novel epidemiological approach, combining bacterial isolation, SNP-based genotyping and global positioning system (GPS) case localization. Our work uncovers the genetic diversity and the corresponding spatio-temporal distribution of *S*. Typhi found within this local population, providing novel insights into the transmission of typhoid in this urban setting. The methodology developed here offers the potential for the design of rational and efficient intervention strategies against typhoid and other infections caused by bacteria with equally limited genetic diversity.

## 3 Material and methods

### 3.1 Ethical approval

This study was conducted according to the principles expressed in the Declaration of Helsinki and was approved by the institutional ethical review boards of Patan Hospital, the Nepal Health Research Council and the Oxford University Tropical Research Ethics Committee (OXTREC). All enrollees were required to provide written informed consent for the collection of samples, residential mapping and subsequent analysis, and in the case of children this was provided by the parent or guardian.

### 3.2 Study site

The site for this study was Lalitpur Sub-Metropolitan City (LSMC) in Kathmandu. LSMC is separated from the metropolitan city of Kathmandu to the north by the Bagmati River, has a land area of 15.43 km^2^ and a population of 162 991 living in 68 922 households, according to the 2001 Nepalese census [26]. Fifty-one per cent of the population is male and the area has a population density of 10 560 people per km^2^ [26]. The location for enrolment to this study was Patan Hospital, a 318-bed government hospital providing emergency and elective inpatient services, fulfilling a primary care service for the local population. It is one of the few locations in LSMC capable of performing a blood culture and an accurate microbiological diagnosis of typhoid. Antimicrobials are available without prescription in the community in a variety of public and private outlets, and there are numerous private physician clinics where patients may seek advice and clinical diagnosis for febrile disease, including typhoid fever. There has been no widespread implementation of a typhoid vaccine in this area. A generic typhoid Vi vaccine is available for purchase in some healthcare settings; however, there is limited community uptake.

### 3.3 Definition of cases

A case of typhoid fever was defined as a patient from whom *S*. Typhi or *S*. Paratyphi A was cultured on arrival in the primary care clinic at the outpatients department according to the methodologies described below. Cases were all attendees at Patan Hospital between June 2005 and May 2009 [18], and were all enrolled in one of three consecutive randomized controlled trials for the treatment of uncomplicated typhoid fever. These trials were gatifloxacin versus cefixime (ISRCTN75784880) [27], gatifloxacin versus chloramphenicol (ISRCTN53258327) [28] and gatifloxacin versus ofloxacin (ISRCTN63006567). The clinical trial enrollees were therefore a proportion of the total typhoid fever cases and a representative proportion of the total uncomplicated typhoid cases within the local population. Eligibility criteria were the same during all the treatment trials and, therefore, across the entire study period considered here. Patients were eligible for enrolment if they had clinically diagnosed typhoid (including a temperature of >38°C), were blood culture-positive for *S*. Typhi or *S*. Paratyphi A, resident within 4 km of the hospital (straight line distance) and aged between 2 and 65 years; eligibility criteria are described in detail by Pandit *et al*. [27].

### 3.4 Definition of controls

A control was defined as an afebrile outpatient attendee of the primary care facility of Patan Hospital seeking medical assistance from within the same radius of the hospital as the cases. These controls were used to adjust the spatial estimate of *S*. Typhi and *S*. Paratyphi A risk for the distribution of the population, and to account for referral bias and locality bias of healthcare-seeking behaviour for outpatients. We recorded the location of the residences of 2048 afebrile patients attending Patan Hospital over a yearlong period from June 2008 to May 2009. The inclusion criteria for this group were: afebrile (to account for typhoid patients with a negative blood culture or other infections that may also cause fever and may have a similar spatial distribution to typhoid, such as leptospirosis or Rickettsial infections [29]), aged between 2 and 65 years, and providing written consent to the mapping of their residence. All outpatients meeting these criteria and attending the primary care facility on a weekday between 0900 and 1200 h were invited for enrolment.

### 3.5 Microbiological culture

Samples of 10 ml of anti-coagulant blood were collected in ethylene diaminetetraacetic acid (EDTA) tubes from febrile patients over the age of 12 years, and 5 ml from those 12 years of age or younger. For the culture of *Salmonella* serovars, 6 and 3 ml of blood were used for those >12 years and ≤12 years of age, respectively. EDTA blood was inoculated into 30–50 ml of medium containing tryptone soya broth and sodium polyethanol sulphonate. The inoculated medium was incubated at 37°C and examined daily for bacterial growth over a 7-day period. If growth was observed, the medium was sub-cultured onto MacConkey agar medium to isolate invasive *Salmonella* serotypes. Any colonies presumptive of *S*. Typhi or *S*. Paratyphi A were identified using standard biochemical tests and serotype-specific antisera (Murex Biotech, Dartford, UK). All *S*. Typhi and *S*. Paratyphi A strains were stored at −80°C in 20 per cent glycerol at Patan Hospital. Duplicates were dispatched for secondary verification at the microbiology laboratory at Oxford University Clinical Research Unit in Ho Chi Minh City and were stored until DNA extraction at −80°C in 20 per cent glycerol.

### 3.6 Geospatial analysis

Addresses are unreliable in this location; consequently, community medical assistants (CMAs) individually recorded the locations of the water spouts and the residences of the patients and controls using a handheld Etrex legend GPS device (Garmin, Southampton, UK). A second member of the study team verified all locations. Multiple infections in the same residence were recorded by a CMA and validated by an additional group member. Multiple household infections were defined as more than one culture positive (*S*. Typhi or *S*. Paratyphi A) infection in a single dwelling. GPS data (in decimal degrees, e.g. 27.67715, 85.32606) were entered along with patient data in Excel 2007 (Microsoft, Redmond, WA). GPS locations were converted to kml format and visualized and validated in Google Earth Pro v. 6.0.3.2197 (http://www.google.com/earth/index.html). GIS data (land usage, hydrology and transport routes) concerning LSMC and the surrounding area were provided by the Ministry of Land Reform and Management, Geodetic Survey Department, Government of Nepal, Kathmandu, Nepal. These data were combined with the GPS location data concerning the cases, controls and water spouts in Quantum GIS v. 1.5.0 (http://www.qgis.org/) and ArcView v. 9.3 (ESRI, Redlands, CA). Distance to nearest water spout of the cases and the controls was calculated in ArcView using a decimal degrees distance calculator.

A water spout is a location within LSMC where the general populace access the ground water for consumption and household use. The historic stone spouts are features throughout the city and are often highly decorated; the water is gravity-dependent, and is replenished through rainfall and snowmelt from the surrounding mountains. Natural soft-rock aquifers act as reservoirs for ground water; ultimately, the untreated water enters the stone spouts from the aquifers through a series of underground channels.

For elevation analysis, a global 90 m digital elevation model dataset, originally developed by the US National Aeronautics and Space Administration, was obtained from the Consultative Group for International Agriculture Research (CGIAR) Consortium for Spatial Information (http://srtm.csi.cgiar.org/). The elevation of each case and control residence was extracted in ArcView. All map figures were created from screenshots of the required datasets in Google Earth Pro v. 6.0.3.2197.

### 3.7 Detection of spatial clustering

The scale and significance of clustering of typhoid fever cases with *S*. Typhi and *S*. Paratyphi A relative to controls were assessed using Ripley's *K*-functions, by the approach of Chetwynd *et al*. [30]. The *K*-function produces a plot of the degree of spatial clustering of the cases relative to the controls over different distances. *K*(*t*) is the number of events within distance *t* of an arbitrary event, divided by the overall density of events. We modelled 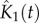 and 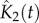, the homogeneous *K*-functions for cases and controls, respectively, and plotted the difference between them over different separating distances, *t*. This gives a plot of the degree of spatial clustering of the cases relative to the controls at different spatial scales. Statistical significance of the observed difference can be assessed using simulation, under the null hypothesis of no spatial clustering. For a series of iterations, the status (case or control) is randomly reallocated to each residence, and the difference between *K*_1_(*t*) and *K*_2_(*t*) is calculated with the simulated dataset. An envelope, showing the maximum and minimum of the simulated values of *K*_1_(*t*) − *K*_2_(*t*) at each separating distance, can be plotted relative to the observed 
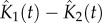
. Significant clustering is deemed to occur over separating distances *t* for which the observed difference between 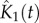 and 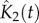 exceeds the simulated envelope. We performed 199 simulations, giving a significance threshold of *p* = 0.05 for rejecting the null hypothesis.

### 3.8 Spatial prediction of risk

Spatial analysis was performed to identify clusters of infections by relating the spatial density of typhoid cases to that of controls, controlling for the distribution of the general population, the effect of elevation and distance to the nearest water spout. Spatial risk prediction was performed in a generalized additive modelling (GAM) framework using an approach reported for case–control data [31]. The models were logistic regression models, where *Y*_*i*_ = 1 for cases and 0 for controls, Elev_*i*_ is the elevation in location *i*, and *P*(*Y*_*i*_ = 1|Elev_*i*_, *S*_*i*_) is given by

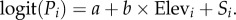


The intercept *a* represents the ratio of cases to controls and *b* is the coefficient for the relationship between elevation and the outcome, logit(*P*_*i*_). We also tested quadratic relationships between elevation and each outcome, and included the quadratic term if it resulted in a lower value of the Akaike's information criterion (AIC). *S*_*i*_, representing residual spatial variation after accounting for the effect of elevation, was modelled using a bivariate smoothing function in the longitudinal and latitudinal planes, using a locally estimated scatterplot smoothing (LOESS) regression smoother. We tested different smoothing bandwidths and selected the optimal bandwidth for each model by minimizing the AIC, as performed by Webster *et al*. [31]. We then used the selected GAM to predict adjusted log odds for each location on a prediction grid that encompassed an area bounded by a convex polygon containing the locations of all cases and controls. We then created a ‘null’ model by omitting the elevation and smoothing terms to provide a reference (equivalent to the ratio of the cases to controls) for calculating odds ratios at each prediction location.

### 3.9 Single-nucleotide polymorphism genotyping

DNA was extracted from *S*. Typhi isolates (electronic supplementary material, table S1) using the Wizard Genomic DNA Extraction Kit (Promega, Fitchburg, WI). The quality and concentration of the DNA were assessed using the Quant-IT Kit (Invitrogen, Carlsbad, CA) prior to SNP typing. Alleles at 113 *S*. Typhi chromosomal loci (electronic supplementary material, table S2) were determined using the iPLEX Gold assay (Sequenom Inc., San Diego, CA). Assays for all SNPs were designed using the MassARRAY assay design software v. 3.1 (Sequenom Inc.). Samples were amplified in multiplexed PCR reactions before allele-specific extension. Allelic discrimination was obtained by analysis with a MassARRAY Analyzer Compact mass spectrometer. Genotypes were automatically assigned and manually confirmed using MassArray TyperAnalyzer software v. 4.0 (Sequenom Inc.). The resulting alleles were used to assign each *S*. Typhi isolate to defined haplotypes or genotypes as described previously [17,20,21].

### 3.10 High-throughput sequencing

Forty *S*. Typhi H58G isolates were arbitrarily selected from the H58G strains with sufficient DNA for additional variant detection by whole genome sequencing (electronic supplementary material, table S2). Index-tagged Illumina libraries were prepared for each sample and sequenced 12 per lane using an Illumina GAII machine, as described previously [32]. Sequencing was successful for 38 of the 40 samples (electronic supplementary material, table S2). The software package Maq [33] was used to align the readings to the reference genome sequence for *S*. Typhi CT18 [34] (EMBL: AL513382) and to identify single nucleotides that differed from the corresponding reference nucleotides (SNPs), as described previously [20]. These SNP loci were compared with those identified previously among 19 *S*. Typhi genomes [20], to confirm that the sequenced isolates had been correctly assigned to the H58G haplotype and to identify SNPs that differentiated among the newly sequenced H58G isolates. A total of 16 such SNPs were identified in two or more sequenced isolates. Alleles at these loci were determined for all study isolates of the H58G haplotype using an iPLEX Gold assay as described above (Sequenom Inc.). The assay succeeded (defined as at least 90% of isolates being assigned to one of the two alleles, and no isolates assigned a heterogeneous genotype) for 13 SNPs. Genotyping data were available for analysis from 387 *S*. Typhi isolates for which there was a GPS location, and the remaining 44 isolates were either unable to be recultured, failed DNA extraction or failed SNP genotyping.

### 3.11 Water sampling and real-time polymerase chain reaction

When permitted by water flow (the municipal water supply to water spouts is dependent on groundwater levels and, therefore, the monsoon), mid-flow water samples were collected weekly over a 1-year period from May 2009 to April 2010 from three municipal water spouts within the region of highest spatial typhoid risk (figure 4*a*). A total of 1.5 l of water was collected from each of the locations on each sampling visit. For microbiological analysis, 20, 50 and 100 µl of undiluted water samples were plated onto Mueller Hinton, XLD and MacConkey agar plates, respectively, and incubated for 18 h at 37°C. Enrichment for *Salmonella* spp. was performed after filtration of 100 ml of water through a 0.45 µm filter (Millipore, Billerica, MA). The filter was removed, placed in 90 ml of soya broth, vortexed and incubated for 18 h at 37°C. After overnight incubation, 1 ml of the pre-enrichment culture was transferred to 10 ml of selenite broth and incubated at 37°C for 24 h. The overnight broth was then plated onto Mueller Hinton, XLD and MacConkey agar plates, and incubated as before. Colony-forming units per millilitre were estimated using a three-tube most probable number (MPN) method, as described previously [35]. For molecular detection of *S*. Typhi and *S*. Paratyphi A in the water samples, total metagenomic DNA was extracted from the remaining matter after 100 µl of water was passed through a 0.45 µm filter using a metagenomic DNA Isolation Kit for water (Epicentre, Madison, WI) according to the manufacturer's recommendations. Purchased filter-sterilized water was used as a negative control for culturing and PCR amplification. Real-time PCR to detect DNA sequences from *S*. Typhi and *S*. Paratyphi A was performed on all metagenomic DNA samples using 10 µl of template DNA, following the methodology described previously [36].

### 3.12 Statistical analysis

The spatial clustering *K*-functions were created using the ‘splancs’ package and GAMs were created using the ‘gam’ package. The logistic regression models were used to assess spatial risk, the mean distance between residences to the nearest water spout and the effect of elevation. All other statistical analyses were performed in R v. 2.9.0. Statistical tests used were: two-sample *t*-tests with equal variance for case–control comparisons of elevation and mean distance between residences to the nearest water spout; the Mantel test with 1000 simulations for comparing spatial, temporal and genetic distances (i.e. Euclidean distance (latitude/longitude) versus temporal distance (days), Euclidean distance versus phylogenetic distance and temporal distance versus phylogenetic distance); *χ*^2^-test for proportional quantification of individual *S*. Typhi haplotypes in a geographically defined area. To test the hypothesis of transmission within a residence, a randomization test was applied to data from the 55 residences with multiple, genotyped *S*. Typhi isolates. Isolates were randomly placed in residences according to the true number observed in each residence and the genotype–frequency distribution of the *S*. Typhi isolates. In each of 1000 randomizations, the number of residences with identical genotypes was recorded, and the resulting distribution was compared with the observation of 11 residences with only identical isolates. Because no temporal–genotype correlation was observed with a Mantel test, there was no requirement for different randomizations for different study periods.

## 4 Results

### 4.1 Geographical clustering of typhoid fever

**Figure 1.**
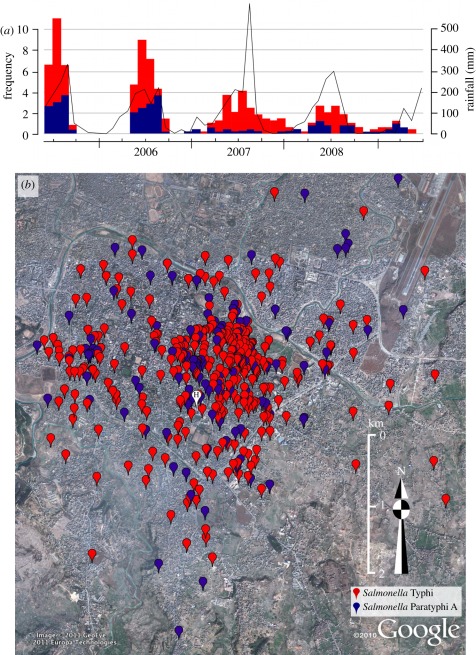
The temporal and spatial distribution of typhoid infections. (*a*) Histogram showing the monthly temporal distribution of *S*. Typhi (red) and *S*. Paratyphi A (blue), and the corresponding monthly rainfall over the 4-year study period. (*b*) Google Earth map, of the study site showing locations of the residences of 584 culture-confirmed typhoid patients: 431 *S*. Typhi (red) and 153 *S*. Paratyphi A (blue). The site of patient enrolment (Patan Hospital) is marked H.

Over a period of 4 years (June 2005 to May 2009), we used handheld GPS devices to locate the residences of 584 culture-confirmed typhoid fever patients—431 (73.8%) infected with *S*. Typhi and 153 (26.2%) with *S*. Paratyphi A. All patients were traced after attending the outpatient department at a single hospital in Kathmandu and were resident within a 4 km radius of this location. The temporal typhoid distribution is shown in figure 1*a*, which follows an annual seasonal trend with the preponderance of infections (420 out of 584, 71.9%) occurring during the monsoon season, between June and September. The primary mapping data showed that typhoid, caused by both *S*. Typhi and *S*. Paratyphi A, was heavily clustered in the northeast of the study area (figure 1*b*).

**Figure 2.**
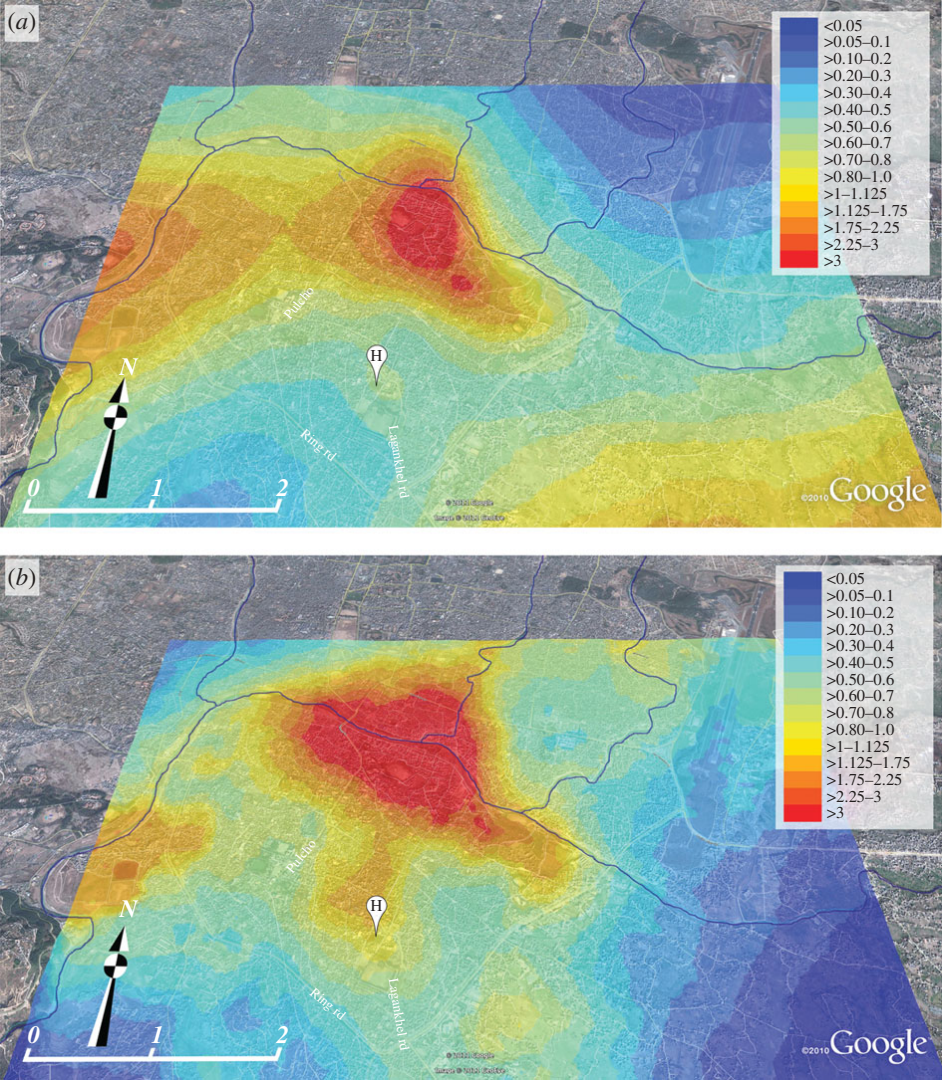
The variable spatial risk of typhoid infections. Elevated Google Earth map of the study terrain with heat map overlays showing the predicted spatial odds for (*a*) *S*. Typhi and (*b*) *S*. Paratyphi A infections compared with controls, as calculated by spatial risk modelling. Spatial odds for typhoid infections are scaled from low (blue) to high (red) as shown by the key. The site of patient enrolment is marked H; the lower-left scale represents distance in kilometres and the route of the Bagmati River is highlighted (flow: west to east).

To assess the scale and significance of the typhoid spatial clustering, we fitted a spatial model using the residences of 2048 afebrile outpatients, resident within the same hospital radius as the cases, to control for population density and hospital referral patterns [30]. The dispersal of typhoid cases was non-random, as both *S*. Typhi and *S*. Paratyphi A cases demonstrated extensive spatial clustering (up to approx. 4.4 km for *S*. Typhi and up to approx. 1.7 km for *S*. Paratyphi A), in comparison with the controls (electronic supplementary material, figure S1). Furthermore, by comparing the spatial distribution with the date of bacterial isolation, we found that spatio-temporal case clustering was evident throughout the study period (*p* = 0.002; Mantel test), consistent with small outbreaks occurring during each monsoon season (figure 1). To identify specific typhoid infection hotspots, we constructed a model to infer the spatial risk for *S*. Typhi and *S*. Paratyphi A infections, correcting for the effects of population density and hospital referral pattern with the afebrile controls (figure 2) [31]. Despite *S*. Paratyphi A being more diffusely distributed than *S*. Typhi, the areas of highest and lowest spatial risk were comparable for both pathogens and demonstrated considerable commonality, yet *S*. Paratyphi A infections were more diffuse than *S*. Typhi infections and seem to be associated with downstream river flow (figure 2). The focal point for typhoid infections was located towards the north of the study area and formed an elongated cluster following the route of the Bagmati River. Within this region of highest spatial typhoid risk, the risk ratio for both *S*. Typhi and *S*. Paratyphi A infections, with respect to the controls, were in excess of 3 : 1 (figure 2).

### 4.2 Genotyping of circulating *Salmonella* Typhi

**Figure 3.**
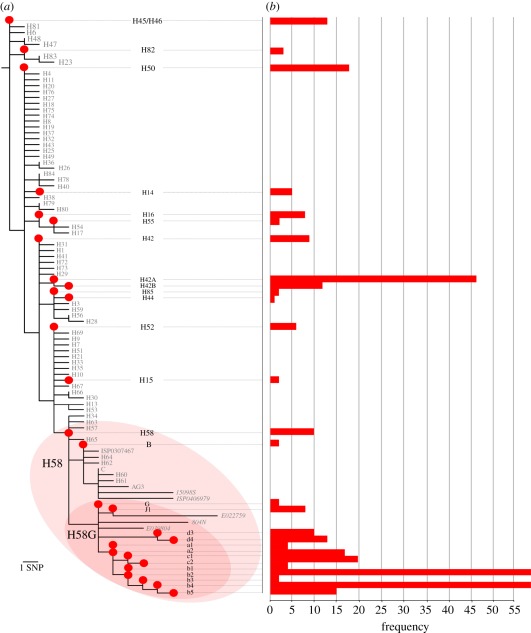
Phylogenetic tree and frequencies of *S*. Typhi genotypes. (*a*) Phylogenetic tree showing the haplotype distribution of 387 *S*. Typhi strains isolated in the study area between June 2005 and May 2009. Red circles and black text indicate genotypes that were detected among the study isolates; grey text indicates genotypes that were defined by assayed loci, but not detected among the study isolates (i.e. would have been detected if present). The H58 and H58G clonal groups are highlighted. (*b*) Horizontal bar plot indicating the frequency of isolation of each *S*. Typhi genotype over the 4-year study period, according to the scale denoted on the *x*-axis.

We have previously shown that SNP-based genotyping is a powerful approach to discriminate within *S*. Typhi populations, providing information on phylogenetic lineage and related phenotypes, such as antimicrobial resistance [21,23]. Further, by resequencing highly related local genotypes, it is possible to define microevolution in real-time by identifying SNP accumulation. Here, we used the Sequenom platform to genotype DNA extracted from 387 local *S*. Typhi (89.8%) with 73 previously identified informative SNP loci (electronic supplementary material, table S1) [21]. This initial typing identified 14 genetically distinct *S*. Typhi clades circulating in this district during the study period (figure 3*a*). However, 68 per cent of the isolates (*n* = 259) were of haplotype H58, which we have shown to be expanding globally and associated with resistance to multiple antimicrobials [17,24]. As H58 represents a highly clonal group, we exploited an additional 38 SNPs earlier identified as discriminating within this clone to reanalyse the 259 H58 isolates [24]. We found that 237 of the H58 *S*. Typhi (92%) belonged to a single sub-group that we have previously named H58G [22]. We hypothesized that the H58G sub-group had undergone expansion during the period of the study, predicting that this dominant group would have accumulated further mutations during its persistence within the local human population, which could be used to further differentiate within the clonal group. Consequently, we selected 40 H58G strains for Illumina whole-genome sequencing and SNP discovery. Using the criterion of a SNP being present in at least two of the 40 H58G isolates, we identified 13 novel SNPs, and screened all H58G isolates with these additional SNPs (electronic supplementary material, figure S2). The resulting phylogeny revealed the expansion of H58G into 3 lineages, the distribution and designations of which are outlined in figure 3*a* and in electronic supplementary material, figure S2. Over the 4-year period of the study, we found 28 different *S*. Typhi genotypes circulating in the study area (figure 3*b*). There were annual fluctuations in the proportions of the various genotypes detected, with some genotypes detected annually through the period of investigation, yet this variability had no obvious pattern (electronic supplementary material, figure S3).

### 4.3 *Salmonella* Typhi genotype distribution within typhoid clusters

**Figure 4.**
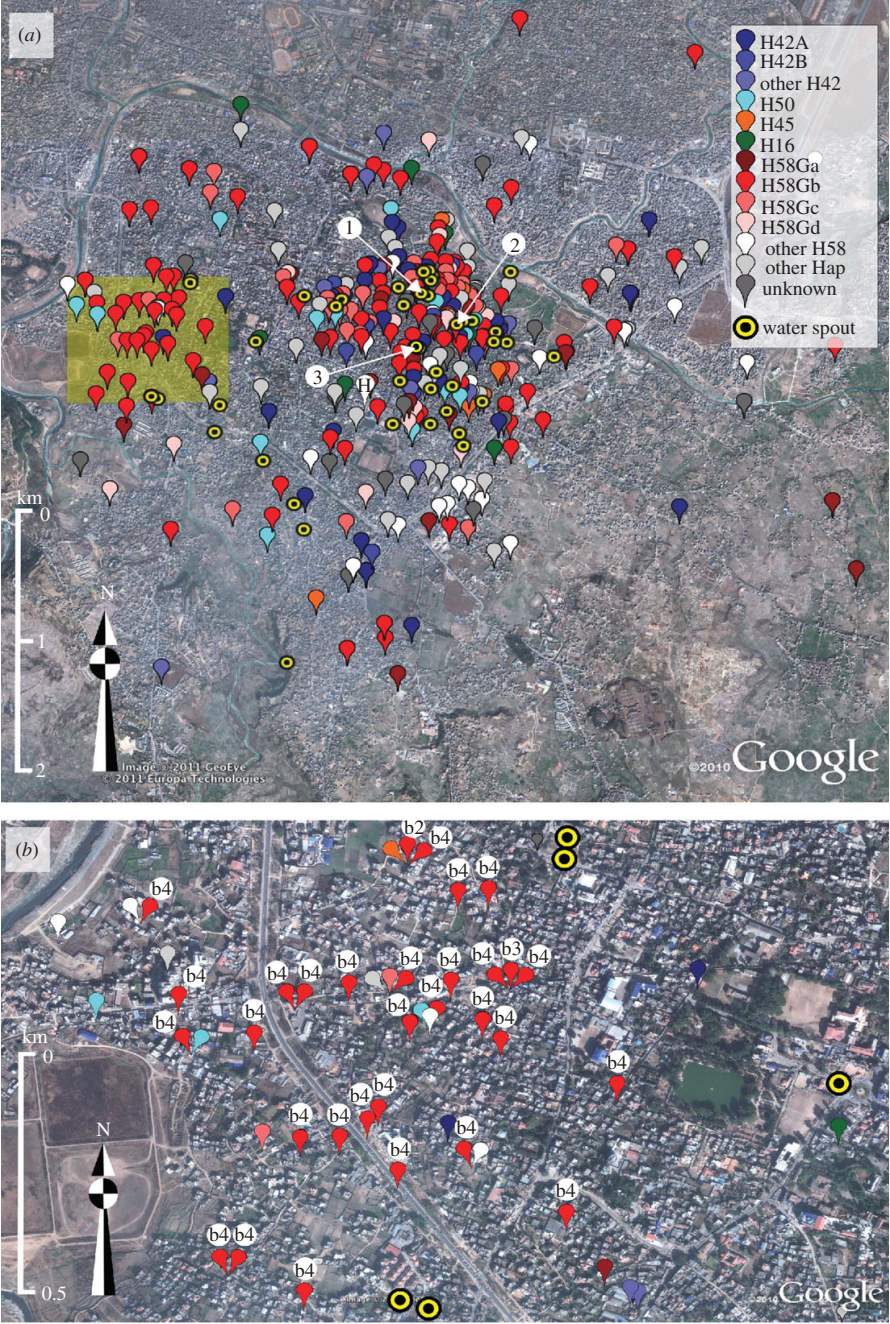
The spatial distribution of *S*. Typhi genotypes. Google Earth maps of the study site showing locations of the residences of culture-confirmed *S*. Typhi infections, categorized by *S*. Typhi genotype (defined in figure 3) and the 42 functional water spouts including the three water-sampling sites (labelled 1, 2 and 3), according to the legend provided. (*a*) All 431 culture-confirmed *S*. Typhi infections from the 4-year study period. The site of patient enrolment is marked H. (*b*) Culture-confirmed *S*. Typhi infections occurring in a concentrated 1 km^2^ cluster to the west of the hospital (shaded as in (*a*)). The subgroups of the H58Gb are shown by the lower-case letters and numerals associated with red markers.

By combining genotype information with GPS location data, we investigated the spatial distribution of the 28 different *S*. Typhi genotypes (figure 4). One would predict that a succession of single-source infections would be temporally and spatially related, and would comprise an individual genotype. We identified a 1 km^2^ cluster of *S*. Typhi cases, located in the northwest region of the study area, which met these criteria (figure 4*b*). Within this cluster, 28 of 39 (71.2%) isolates in year 1 were of identical genotype (H58G-b4). In subsequent years, the number of *S*. Typhi cases in this area decreased (7–13 cases per year; *p* = 0.03; *χ*^2^-test), as did the contribution of *S*. Typhi H58G-b4 (two to six cases per year; *p* < 0.0001; *χ*^2^-test), suggesting an isolated single-genotype outbreak in year 1. However, this small genotype cluster was exceptional, as we could find no other clustering of individual genotypes. In fact, despite spatial and temporal typhoid case clustering, the distribution of *S*. Typhi genotypes was ostensibly random (figure 4). Case clusters occurring over limited time periods were composed of multiple genotypes and there was no overall evidence of spatial (*p* = 0.61; Mantel test) or temporal (*p* = 1; Mantel test) genotype clustering. These data indicate an overwhelming contribution of indirect transmission, indicative of exposure to diversely contaminated material, as opposed to person-to-person transmission of individual organisms.

Waterborne transmission of typhoid has been demonstrated in several epidemic settings [37,38], and we have previously hypothesized that the local municipal water supply may be an important vehicle for typhoid in the study location [18]. Using GPS devices, we located the sites of the 42 functional municipal water spouts in the study area (figure 4) [39] and used the spatial risk model to assess the effects of water spout proximity and elevation on typhoid risk. Water spout proximity was significantly associated with the risk of typhoid caused by both *S*. Typhi (OR 0.48, 95% CI (0.37, 0.62), *p* < 0.0001) and *S*. Paratyphi A (OR 0.60, 95% CI (0.40, 0.88), *p* = 0.009). Lower elevation was also significantly associated with typhoid risk, as the mean elevation of *S*. Typhi and *S*. Paratyphi A patient residences were 3.27 and 3.78 m lower, respectively, than those of afebrile controls (OR 0.83, 95% CI (0.77, 0.90), *p* < 0.0001 for *S*. Typhi; OR 0.83 95% CI (0.75, 0.93), *p* = 0.001 for *S*. Paratyphi A).

To investigate whether the municipal water supply was contaminated with the agents of typhoid, when permitted by seasonal water flow over a 1-year period from May 2009 to April 2010, we collected weekly water samples from three water spouts located within the region of highest spatial risk (118 samples; figure 4). Using conventional microbiological coliform culture methods, we found consistent faecal contamination, ranging from 3.3 to 2.4 × 10^6^ cfu ml^−1^, yet we were unable to detect either *S*. Typhi or *S*. Paratyphi A after selective culturing. However, using a real-time PCR assay we previously developed to identify *S*. Typhi and *S*. Paratyphi A in biological specimens [36], we detected the presence of DNA sequences specific for *S*. Typhi and *S*. Paratyphi A in the majority of water samples from all three locations. Out of 118 metagenomic DNA preparations from filter surfaces, 101 (85.6%) were positive for *S*. Typhi, 91 (77.1%) were positive for *S*. Paratyphi A and 77 (65.3.%) were positive for both *S*. Typhi and *S*. Paratyphi A. There was some evidence of a relationship between seasonality and water samples testing positive for the agents of typhoid, as more samples were positive during the monsoon season. However, it is difficult to draw conclusions regarding seasonal contamination, as the water spouts generally only produce flowing water when the aquifers are filled as a result of rain water replenishment.

### 4.4 Intra-household typhoid transmission

**Figure 5.**
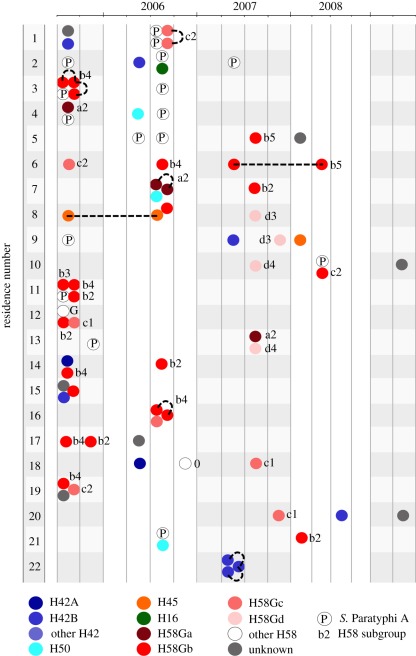
Intra-residence typhoid infections. Illustration depicts the 22 residences (vertical axis) with three or more culture-confirmed typhoid infections over the period of investigation (horizontal axis). Each individual infection is shown by coloured circles, which are grouped into three-month periods. Colours indicate the *Salmonella* serotype (the letter P indicates an *S*. Paratyphi A infection) or *S*. Typhi genotype associated with each infection, according to the legend provided (as defined in figure 3). Lower-case letters and numerals associated with red circles refer to the individual H58G subgroup (defined in figure 3). Broken black lines link isolates of the same genotype within a single residence.

We were able to explore potential transmission routes in even finer detail as, over the 4-year period, multiple residences had more than one case of typhoid (figure 5). Thirty-seven residences (43%) had both *S*. Typhi and *S*. Paratyphi A infections, and 59 had multiple *S*. Typhi infections. Genotype data were available for more than one *S*. Typhi in 55 of these households; of these, 44 (80%) households had infections caused by distinct genotypes and 11 had infections caused by identical *S*. Typhi genotype only. The observed number of intra-household *S*. Typhi pairs of identical genotype was greater than that expected by chance (*p* = 0.027; randomization test), providing evidence of direct transmission within individual residences. For paired *S*. Typhi isolated in the same residence within a week, 44 per cent (7 of 16) shared the same genotype, consistent with direct person-to-person transmission during an acute infection. However, multiple genotypes within individual residences were more commonly observed (figure 5). Given multiple cases of typhoid in a single residence, the odds of the infections being caused by distinct organisms as opposed to an identical genotype were in excess of 3 : 1.

## 5 Discussion

Technological limitations in molecular microbiology have previously hindered our ability to distinguish between low-diversity organisms, and consequently to study the local ecology and transmission patterns of bacterial pathogens. However, the resolution now permitted by high-throughput sequencing and SNP analysis has facilitated an enhanced understanding of the global population of *S*. Typhi and other bacterial pathogens with limited genetic diversity [17,32,40]. Here, for the first time, we have combined elements of classical epidemiology with high-throughput sequence analysis and GPS-based spatial analyses to longitudinally study the local distribution and infer the transmission of a human-restricted bacterial pathogen in the field. Using these methods, we have heightened the knowledge of typhoid transmission in a densely populated, highly endemic urban area.

Our approach demonstrated widespread disparity in the spatial risk of typhoid in this setting. Specifically, we found extensive clustering of typhoid infections in particular locations, the main regions of which were comparable for both *S*. Typhi and *S*. Paratyphi A. Yet *S*. Paratyphi A infections were more diffuse than *S*. Typhi infections, and associated with the Bagmati River. These data indicate that, overall, both pathogens exhibit a similar spatial risk and transmission pattern, with some evidence that *S.* Paratyphi A infection risk spreads downstream from the main focal point. We suggest that *S*. Typhi infections are associated with a limited number of specific locations, whereas *S*. Paratyphi A may have an enhanced ability to disseminate, potentially by contaminating ground water via the Bagmati River. Typhoid case clustering has been observed in other urban settings, drawing associations with population density, overcrowding and hygiene practices [12,41]. Yet here, spatial case clustering of typhoid was unrelated to local population density. This is somewhat counterintuitive for a human-restricted pathogen and suggests local variability in risk factors for infection. The transmission of typhoid is often linked with poor sanitation, contaminated water and contact with an acute case or carrier [13,42–44]. Our data strongly suggest that indirect transmission is dominant in this setting, which is substantiated by the association of typhoid cases with low elevation and water spout proximity. A comparable argument linking *S*. Typhi isolated during a typhoid epidemic in Chile to transmission via faecal-contaminated water supplies has been made previously [45]. Here, we propose that people living near to water spouts are of greater dependence on these for household water, and typhoid incidence is likely to be associated with faecal contamination of ground water during the monsoon, particularly in areas with low elevation. These two variables (elevation and water spout proximity) are likely to be interconnected, as the water is gravity-dependent and the water spouts are more common in low-lying zones. Our year-long water sampling supports this hypothesis, as we found that the majority of water samples had exceptionally high levels of faecal contamination, and *S*. Typhi and *S*. Paratyphi A nucleic acid could be detected in the majority of samples. Our inability to directly culture *S*. Typhi and *S*. Paratyphi A, despite molecular evidence of their presence, warrants additional investigation. These organisms are notoriously furtive in the environment, and *S*. Typhi culturing from water sources has been reported rarely, and only in the context of single-source outbreaks [37,38]. We suggest, as has been proposed before, that either the organisms are in a quiescent form and do not respond after exposure to rich microbiological media [46], or, as a result of extensive faecal contamination, they were simply overwhelmed by other less fastidious organisms.

We aimed to differentiate the *S.* Typhi population to infer potential transmission patterns. Yet, our initial genotyping screen found that 68 per cent of all local *S*. Typhi isolates belonged to a single genotype, H58. The local dominance of these H58 strains is largely unsurprising as the H58 group now prevails globally as a result of recent clonal expansion in parts of Asia and Africa [17,23,24]. The augmentation of SNP typing with additional H58-specific loci and the sequencing of selected H58G *S*. Typhi isolates from the study provided enhanced resolution and revealed ongoing local microevolution within the H58 group. Therefore, longitudinally, the *S*. Typhi population was mainly composed of members of the H58 group, intermingled with multiple, less frequently isolated genotypes.

Ultimately, we were able to describe 28 distinct *S*. Typhi genotypes circulating in varying annual proportions in the study area over the 4-year period. This degree of genetic diversity was sufficient to dissect potential spatial and temporal relationships between the circulating *S*. Typhi. One would predict that proximal cases occurring as a result of direct transmission from a single source, such as a chronic or acute shedder, would be indistinguishable by SNP typing. However, with the exception of one localized area, we found no temporal or spatial association of individual genotypes and no evidence of local clonal replacement. In fact, the overall pattern of strain distribution was one of erratic, random genotype fluctuation rather than localized genotype clustering. This is indicative of exposure to a wide array of genotypes, rather than direct transmission of the same organism in close proximity. We could find evidence of direct transmission through the isolation of identical genotypes in individual households at a greater rate than would be expected by chance. Yet even within an individual household, multiple cases were more frequently caused by an array of organisms, indicating potential acquisition from outside the residence. One caveat of these findings is that our analysis assumes that patients are infected exclusively with one bacterial genotype. Conversely, owing to SNP discovery bias, it is likely that SNP typing underestimates diversity within a dwelling, as it is possible for two isolates to be identical at all assayed loci despite being genetically distinct at unassayed loci [47]. We have previously demonstrated the presence of *S*. Typhi and *S*. Paratyphi A carriers in this local community [48], yet their precise role in transmission remains unclear. Our data are not consistent with such people playing a prevailing role in direct person-to-person transmission. Rather, we suggest that in this urban typhoid endemic environment, asymptomatic carriers are essential for sustaining a diverse *S*. Typhi population from year to year, providing individual isolated reservoirs for the long-term persistence of a wide array of genotypes which are continually shed into the environment, providing consistent low-level contamination of local water supplies.

We advocate that, in settings such as Kathmandu, improvements in infrastructure are fundamental to the control and elimination of typhoid. Poor water quality, sanitary conditions and the presence of carriers are likely to facilitate the continued persistence of the organisms in the community long after the limited window of immunity induced by the current polysaccharide vaccine [49,50]. Without integrating infrastructural improvements alongside other control measures (diagnosis, treatment and vaccination), we are sceptical as to whether typhoid can be adequately controlled in the long term in such settings. Indeed, this would be consistent with the historical elimination of typhoid from many countries where intervention strategies have invariably been coupled with economic development and improvements in infrastructure.

We have combined high-resolution SNP-based bacterial genotyping with local GPS location data for the first time. Our work provides an enhanced understanding of the ecology and transmission of the agents of typhoid in an endemic setting, and represents an integrated, novel approach to molecular epidemiology that may be directly applied to other diseases caused by other monophyletic or emerging bacterial pathogens.
